# The Critical Role of Partially Exposed N-Terminal Valine Residue in Stabilizing GH10 Xylanase from *Bacillus sp.*NG-27 under Poly-Extreme Conditions

**DOI:** 10.1371/journal.pone.0003063

**Published:** 2008-08-26

**Authors:** Amit Bharadwaj, Sadhu Leelavathi, Sudeshna Mazumdar-Leighton, Amit Ghosh, Suryanarayanarao Ramakumar, Vanga S. Reddy

**Affiliations:** 1 International Centre for Genetic Engineering and Biotechnology, Aruna Asaf Ali Marg, New Delhi, India; 2 Department of Botany, University of Delhi, Delhi, India; 3 Indian Institute of Advanced Research, Gandhinagar, India; 4 Department of Physics, Indian Institute of Science, Bangalore, India; 5 Bioinformatics Centre, Indian Institute of Science, Bangalore, India; Cairo University, Egypt

## Abstract

**Background:**

Understanding the mechanisms that govern protein stability under poly-extreme conditions continues to be a major challenge. Xylanase (BSX) from *Bacillus sp.* NG-27, which has a TIM-barrel structure, shows optimum activity at high temperature and alkaline pH, and is resistant to denaturation by SDS and degradation by proteinase K. A comparative circular dichroism analysis was performed on native BSX and a recombinant BSX (R-BSX) with just one additional methionine resulting from the start codon. The results of this analysis revealed the role of the partially exposed N-terminus in the unfolding of BSX in response to an increase in temperature.

**Methodology:**

We investigated the poly-extremophilicity of BSX to deduce the structural features responsible for its stability under one set of conditions, in order to gain information about its stability in other extreme conditions. To systematically address the role of the partially exposed N-terminus in BSX stability, a series of mutants was generated in which the first hydrophobic residue, valine (Val1), was either deleted or substituted with various amino acids. Each mutant was subsequently analyzed for its thermal, SDS and proteinase K stability in comparison to native BSX.

**Conclusions:**

A single conversion of Val1 to glycine (Gly) changed R-BSX from being thermo- and alkali- stable and proteinase K and SDS resistant, to being thermolabile and proteinase K-, alkali- and SDS- sensitive. This result provided insight into the structure-function relationships of BSX under poly-extreme conditions. Molecular, biochemical and structural data revealed that the poly-extremophilicity of BSX is governed by a partially exposed N-terminus through hydrophobic interactions. Such hitherto unidentified N-terminal hydrophobic interactions may play a similar role in other proteins, especially those with TIM-barrel structures. The results of the present study are therefore of major significance for protein folding and protein engineering.

## Introduction

Understanding the mechanism of protein stability under poly-extreme conditions such as high temperatures, a wide range of pH and resistance to degradation by proteases is a great challenge. Many studies have indicated that there is no single and unique structural requirement for making a protein stable under a variety of extreme conditions; several factors such as increased hydrophobic and aromatic contacts, electrostatic interactions and side chain packing [1] appear to play crucial roles in protein stability. However, the mechanism by which proteins attain the stability to function under poly-extreme conditions remains elusive.

The mutation approach has been extensively used to pinpoint specific interactions that contribute to the stability of various proteins. Studies involving T4 lysozyme and barnase from *Bacillus amyloliquefaciens* as ‘model’ enzyme systems show that many mutations can be stabilizing, destabilizing or without effect [2], [3]. Generally, the surface residues of a protein are widely regarded to be tolerant to substitution, because exposed sites remain exposed in both native and denatured states. However, several studies have shown that the substitution of an amino acid(s) on the protein surface have different effects on its stability, depending on the environment of the mutation site(s) [4]. In contrast to the destabilizing effect of substituting hydrophobic amino acids at the hyper-exposed site on the protein surface [5], such a substitution on the surface of globular proteins has been reported to increase the stability of these proteins [4], [6], [7]. Most importantly, it has been shown that a single amino acid substitution can have vastly different effects on the stability of a protein depending on the location of the mutation within the structure [8].

Xylanases (EC 3.2.1.8) catalyze the hydrolysis of β-1,4 bonds of xylan backbones, the major hemicellulose component of the plant cell wall [9]. Xylanases have several industrial applications including animal feed, bakery, and paper pulp industries. Recently, its use in bioethanol production has gained popularity [10]. Previously, we reported the isolation and characterization of a gene coding for a ∼41 kDa extracellular xylanase from *Bacillus sp.* NG-27 (BSX, 11). BSX is optimally active at a temperature of 70°C (thermostable) and at pH 8.5 (alkali-stable) [10], [11]. BSX does not contain any cysteine residues, which rules out the role of disulfide bridge(s) in its stability under poly-extreme conditions. In the crystal structures of native (2F8Q) and xylosaccharide-bound BSX (2FGL), we were able to identify several structural features important for its alkaline and halophilic stability [12], [13]. BSX has a TIM-barrel structure, which is the most common folding pattern among protein catalysts and is present in approximately 10% of all known enzyme structures. Structural analysis revealed that the N-terminus of mature BSX has Val, Gln, Pro, Phe and Trp as partially exposed hydrophobic residues at positions 1, 2, 3, 4 & 6, respectively. We carried out a comparative circular dichroism (CD) analysis of native BSX and a recombinant BSX (R-BSX) with an additional methionine (Met) resulting from the start codon. Our result provided the first clue about the role of a partially exposed N-terminus on the unfolding of this protein in response to increases in temperature. This led us to explore the structural features that could be crucial for the stability of BSX under poly-extreme conditions. Such knowledge would allow for the modulation of BSX activity to make it more suitable for application in diverse fields such as the paper pulp, animal feed, bakery and biofuel production industries. In addition, this knowledge would provide insight into structure-function relationships under poly-extreme conditions that may be generally applicable to other proteins possessing similar TIM-barrel structures.

To this end, the role of the partially exposed first valine (Val1) in the stability of BSX under poly-extreme conditions was examined by creating a series of mutant proteins in which Val1 was either deleted or replaced with other amino acids with different side chains. All mutant proteins were critically evaluated for their structural, molecular and biochemical properties in comparison to those of native BSX. Our results demonstrate that the N-terminal hydrophobic interactions are crucial for the compact packing and stabilization of the TIM barrel (β/α)_8_ fold, which is required for protein function. To the best of our knowledge, this is the first report of a major role for a single N-terminal amino acid in the correct folding and stability of GH10 xylanase under unrelated extreme conditions such as thermal and alkaline stability, and resistance to SDS and proteinase K treatments.

## Results

### Additional processing of the N-terminus occurs during the secretion of BSX

**Figure 1:**
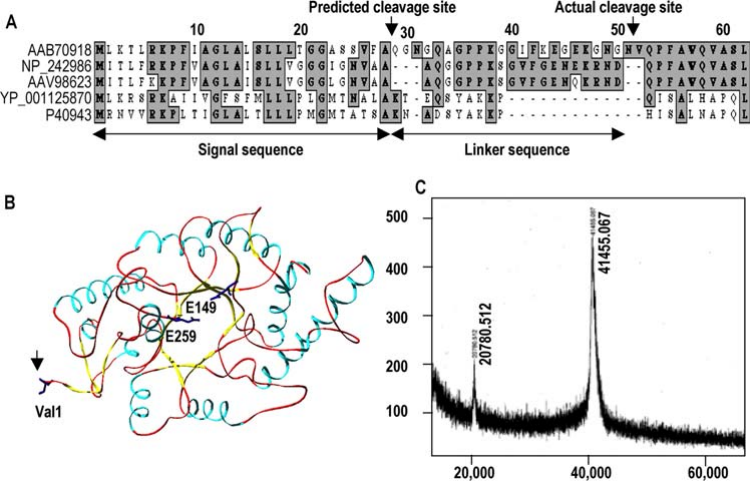
Cleavage site of BSX signal sequence during extra-cellular secretion in *Bacillus Sp.* NG-27. (A) Multiple sequence alignments of the BSX signal peptide with other GH10 xylanases of *Bacillus* origin. AAB70918: *Bacillus sp.* NG-27, used in the present study, NP_242986: *Bacillus halodurans* C-125, AAV98623: *Bacillus halodurans* S7, YP_001125870: *Geobacillus thermodenitrificans* NG80-2, P40943: *Bacillus stearothermophilus* T-6. (B) Ribbon diagram of BSX showing catalytic residues (E149 and E259) and the N-terminus Val1 residue (indicated by arrow). (C) Mass Spectrometry analysis of purified BSX confirming the processing of the first 51 amino acid-long signal sequence during its secretion instead of the predicted 28 amino acids. The predicted signal sequence and the additional linker sequence that is also processed are indicated by the double-headed arrows in (A).

The alignment of the BSX amino acid sequence with those of xylanases from other bacterial sources indicated the presence of a well-conserved secretory sequence of 28 amino acids at the N-terminus (Figure 1A). The crystal structure revealed that the first amino acid of the mature BSX is Val, which appears at position 52 in the amino acid sequence (Figure 1B) [13]. Therefore, in order to determine the correct cleavage site, we sequenced the N-terminus of the enzyme and found that Val52 was indeed the first amino acid. Mass spectrometry analysis showed that the total mass of the BSX was 41.45 kDa, which suggests that during its extracellular translocation, the enzyme is not cleaved after the predicted 28 amino acid signal sequence, but after 51 amino acids (Figure 1C). Taken together, the results clearly indicate that in addition to the predicted signal sequence, an additional 23 amino acids, called the “linker sequence” is removed from the N-terminus of BSX before its secretion from *Bacillus subtilis*.

### Recombinant BSX with an additional Met is more compactly packed than native BSX

**Figure 2:**
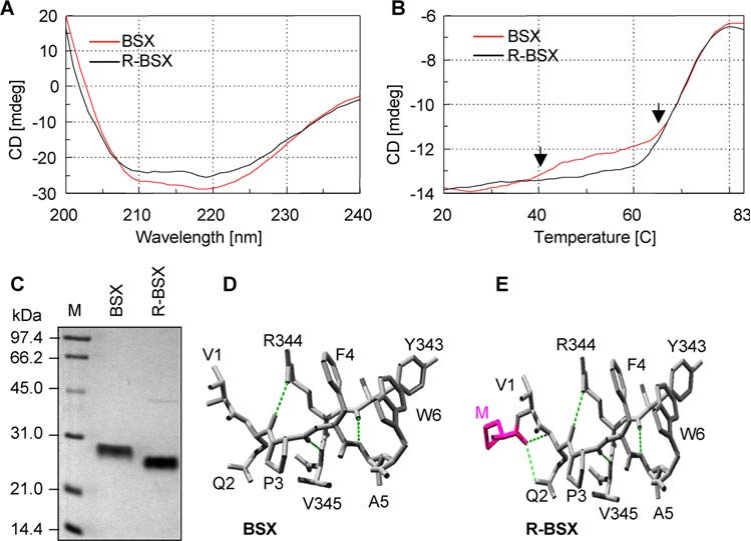
Comparison of BSX and R-BSX structures using CD and SDS-PAGE. (A) Far UV spectra of BSX and R-BSX. (B) CD spectra of thermal denaturation of BSX and R-BSX monitored at 222 nm with a temperature slope of 1°C/min. Note the difference in the thermal unfolding pattern between 40°C–60°C, marked by arrows. (C) SDS-PAGE analysis of BSX and R-BSX, showing higher mobility of R-BSX. To preserve their native conformations, samples were loaded onto the gel without prior boiling. (D) SwissPdb-generated structural models showing the N-terminal region of BSX and R-BSX. Additional hydrogen bonds between Met (start codon) and Gln2 are highlighted by colored dotted lines.

The secondary structures of native (BSX) and recombinant (R-BSX) xylanases were determined by far UV-circular dichroism (CD) with a wavelength range of 190–250 nm. BSX and R-BSX produced identical spectra with two separate negative peaks at 208 nm and 222 nm, indicating the presence of α-helices (Figure 2A). Hence, thermal unfolding of BSX and R-BSX was carried out at 222 nm in a temperature range of 20°C–85°C with a slope of 1°C/min. A careful observation of the thermal unfolding curve of BSX revealed the presence of two structural transitions (Figure 2B). The first was a minor transition at 45°C, followed by a major transition at 60°C, and the protein eventually unfolded completely at 80°C. In contrast, a minor transition at 45°C was completely lacking for R-BSX. The transition at 60°C was almost identical for both BSX and R-BSX (Figure 2B). The early thermal unfolding of BSX could be a result of the early unfolding of the N-terminal region. The lack of thermal unfolding at 45°C in R-BSX indicates that the molecule is more compactly packed at the N-terminal region. In addition to CD analysis, BSX and R-BSX were analyzed on an SDS-PAGE gel without boiling the samples prior to loading in order to observe differences the compactness of the molecules. Here, the mobility of R-BSX was found to be more than that of BSX in spite of the slight increase in the molecular weight of R-BSX due to the presence of an additional Met residue at the N-terminus (Figure 2C). These results indicate that the additional hydrophobic Met residue might be responsible for better packing of the N-terminal region in R-BSX, and reveal the important role of this region in BSX unfolding at higher temperatures, as well as under other extreme conditions.

### Effect of deletion/substitution of Val1 on the unfolding of BSX

**Figure 3:**
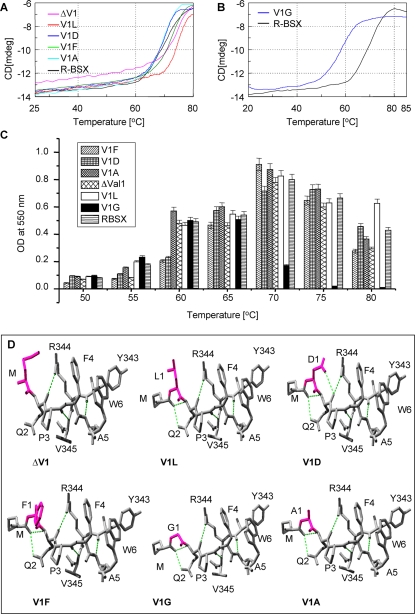
Thermal denaturation and structural features of the R-BSX mutants. (A) CD spectra of thermal denaturation of ΔV1, V1L, V1D, V1F, V1A and R-BSX monitored at 222 nm with a temperature slope of 1°C/min. (B) CD spectra of thermal denaturation of V1G and R-BSX, showing the ∼12°C decrease in T_m_ for V1G. (C) Xylanase activity profile of R-BSX mutants at various temperatures. Maximum activity for V1G was observed at 60°C, differing from other mutants which showed maximum activity at 70°C similar to that of R-BSX. (D) SwissPdb-generated structural models showing the N-terminal region of all R-BSX variants. The amino acid substituted for Val1 is shown in pink. The additional hydrogen bonds resulting from a deletion/substitution are shown in green dotted lines.

To explore whether replacing Val1 with residues that are less or more hydrophobic could alter the folding of the enzyme and in turn alter SDS, proteinase and thermal stability, R-BSX was subjected to site-directed mutagenesis studies. The various deletion and substitution mutants created for this purpose along with the differences in their T_m_ values are shown Table 1. There was no significant difference in the thermal unfolding curves of V1A, V1D and V1F (Figure 3A) compared to that of R-BSX; consequently, the Tm of these mutants were close to that of R-BSX. However, the thermal unfolding curves of V1A and V1D suggest that these mutants unfold completely at 75°C in contrast to R-BSX, which attains complete unfolding at 80°C. Another significant change was observed when Val1 was changed to Leu; unfolding started at 70°C and the enzyme unfolded completely at 80°C (Figure 3A). The slope of the thermal unfolding curve of V1L is ∼10°C steeper than that of R-BSX and the rest of the mutants, indicating that V1L displays maximum co-operativity in its unfolding pathway compared to the other proteins. The thermal unfolding curve of the deletion mutant of Val1 (ΔV1) with a Met (start codon) residue at the N-terminus demonstrates a gradual but continuous decrease in CD signal until 60°C, the temperature at which actual unfolding began (Figure 3A). This continuous decrease in CD signal before 60°C did not give rise to the minor transition as observed in the case of BSX at 45°C, which verified the role of the extra hydrophobic Met residue in providing additional stability to the R-BSX in a temperature range of 45–60°C. The T_m_ of V1G, as calculated from the thermal unfolding curve is ∼58°C, which is 12°C lower than that of R-BSX (Figure 3B). V1G started unfolding at 50°C and completely unfolded at 65°C. It is evident from the thermal unfolding profiles of all the R-BSX mutants that the V1G mutant has the highest sensitivity to temperature, and hence is the most thermolabile mutant created in this study.

**Table 1:** Characteristic features of BSX and its mutants.

S. No.	Protein	Amino acid substitution	N-terminal sequence	Effect on T_m_	SDS stability	Proteinase K stability	Optimum temperature for activity
1	BSX	_	VQPFA	_	Resistant	Resistant	70°C
2	R-BSX	Additional Met	MVQPFA	_	Resistant	Resistant	70°C
3	ΔV1	Val1-	MQPFA	No effect	Resistant	Resistant	70°C
4	V1D	Val1→Asp	MDQPFA	No effect	Resistant	Resistant	70°C
5	V1F	Val1→Phe	MFQPFA	No effect	Resistant	Resistant	70°C
6	V1A	Val1→Ala	MAQPFA	Decreased by ∼2°C	Resistant	Resistant	70°C
7	V1G	Val1→Gly	MGQPFA	Decreased by ∼12°C	Susceptible	Susceptible	60°C
8	V1L	Val1→Leu	MLQPFA	Increased by ∼5°C	Resistant	Resistant	70°C

### Thermal inactivation of xylanase activity

The recombinant R-BSX was expressed as a soluble protein in *E. coli* and showed almost identical enzymatic activity to native BSX (data not shown). Optimum temperatures for the enzymatic activity of R-BSX and the mutants were determined by measuring the xylanase activity at various temperatures. All the deletion/substitution mutants, except V1G, were found to exhibit maximum activity at 70°C (Figure 3C); maximum xylanase activity of V1G was observed at temperatures between 60°C–65°C. This mutant enzyme also had less than 20% activity at 70°C (Figure 3C), consistent with the CD thermal unfolding data. In light of this, all the activity assays for V1G were carried out at 60°C. An important observation was that the V1L mutant showed the highest residual activity at 80°C when compared to other mutants, including R-BSX.

### Structural modeling of Val1 mutants using SwissPdb viewer

Structural models of BSX, R-BSX and the mutants were generated using SwissPdb Viewer [14] by deletion and substitution of Val1 with Phe, Asp, Ala, Leu and Gly in R-BSX (Figure 2D-E and Figure 3D). The R-BSX structural model showed that the main chain oxygen of Met contributes to making two extra hydrogen bonds with the main chain and side chain of Gln2 (Figure 2E). For the V1D mutant, one additional side chain-main chain hydrogen bond was predicted between Asp1 and Gln2. In addition, Asp1 can also form ionic interactions with Arg344, about ∼4.5Å away (Figure 3D). In the case of the V1F mutant, one cation-pi interaction was predicted between Phe1 and Arg344. These additional interactions may provide extra rigidity to V1F and V1D against SDS. No such interaction could be predicted for the V1L mutant; better packing of Leu1 at this position due to its longer side chain might be responsible for its extra thermal stability and the co-operativity in its unfolding. It also appears that the shortening of the side chain in V1A and V1G mutants may be one of the major reasons for the decrease in their stability, as this would expose the core of the molecule to a greater extent.

### Native PAGE analysis of thermal unfolding

**Figure 4:**
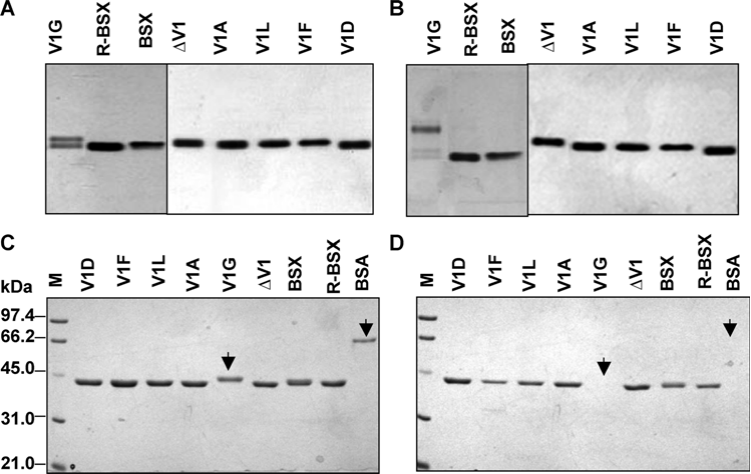
Native PAGE profiles of BSX and R-BSX. (A) and (B) show native-PAGE profiles of thermal unfolding of proteins at room temperature and at 60°C, respectively. V1G was found to unfold after incubation at 60°C for 15 min, whereas no significant unfolding was observed for all the other mutants. Note the slightly faster mobility of R-BSX when compared to BSX, reconfirming the results obtained in Figure 2C. (C) and (D) show SDS-PAGE profiles of all the proteins in the absence and presence of proteinase K, respectively. Note that all proteins except V1G and BSA (indicated by arrow) showed proteinase K resistance.

Native PAGE analysis provides an elegant way to visualize the thermal unfolding of proteins. Therefore, native PAGE was used to monitor the thermal unfolding of BSX, R-BSX and its mutants at various temperatures. Protein samples were incubated at various temperatures for 15 min and then kept on ice before being subjected to native PAGE. Figures 4A and 4B show the native gel profiles of all samples which were incubated at room temperature (25°C) and at 60°C, before native PAGE analysis. It was observed that the V1G mutant migrated as a doublet (Figure 4A), whereas other mutants as well as the R-BSX migrated as single bands when samples were incubated at 25°C. The V1G mutant unfolded almost completely at 60°C (Figure 4B), resulting in its slower migration compared to R-BSX. In contrast, there was no significant change in the migration of other mutants. It should be noted that the results obtained from native PAGE analysis of thermal unfolding were found to be in good agreement with the CD thermal unfolding data, as well as the profiles of thermal inactivation of xylanase activity.

### Effect of deletion/substitution of Val1 on protease stability

The stability of R-BSX and its mutants against proteolytic degradation was assessed by treating the samples with proteinase K overnight at room temperature, followed by SDS-PAGE. All the mutants except V1G were found to be resistant to proteinase K digestion (Figure 4D). In the case of V1G, the protein band disappeared completely after overnight treatment with proteinase K. These results showed that the conformation of the N-terminus in V1G renders it susceptible to protease digestion. Figure 4C shows the SDS-PAGE profile of the protein samples without protease treatment, for direct comparison.

### Effect of deletion/substitution of Val1 on SDS and alkaline stability

**Figure 5:**
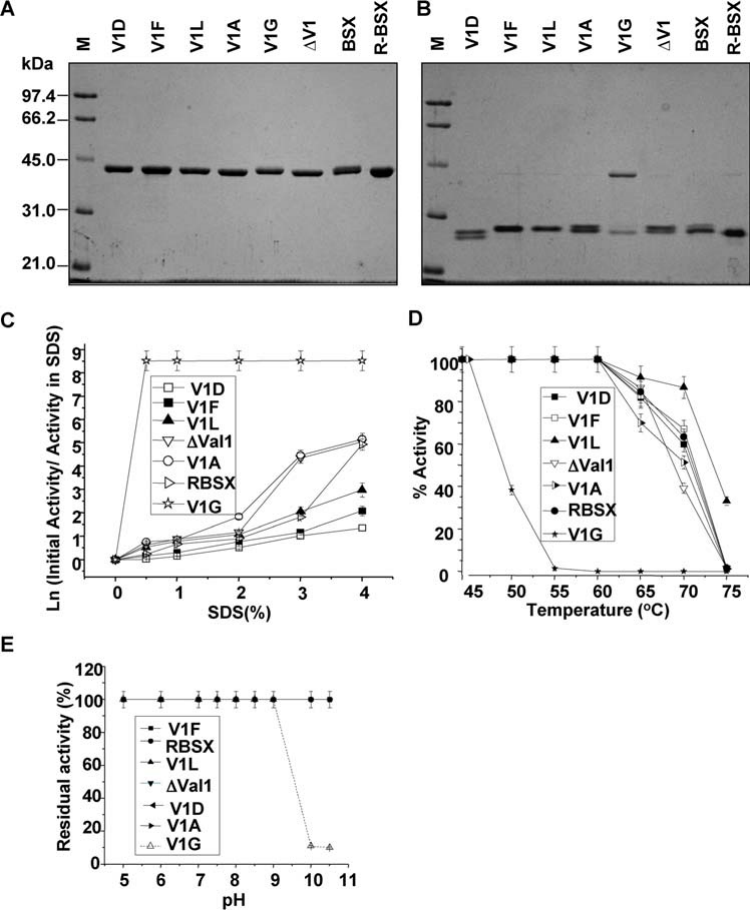
Effect of SDS and proteinase K on the BSX and R-BSX. (A) and (B) show SDS-PAGE profiles for determining the SDS-stability of proteins. Identical protein samples were loaded onto the gel with or without prior boiling for 10 min, respectively. (C) Xylanase activity in the presence of SDS. Samples were incubated for 12 h in the presence of various concentrations of SDS prior to the activity assay. Note that V1F and V1D displayed maximum activity. (D) Thermostability of xylanase and its mutants. Samples were incubated for 15 min at various temperatures and assayed for residual xylanase activity at their respective optimum temperature (see material and methods). (E) Alkaline stability of BSX, R-BSX and its mutants. Note the drastic reduction in the activity of the V1G mutant when the enzyme was incubated above pH 10 prior to the assay.

The SDS-Protein complexes formed when the samples were boiled before loading the gel migrated to their expected positions in the gel based on their molecular weights (Figure 5A). When loaded without prior boiling, R-BSX and all mutant proteins except V1G showed higher mobilities than would be expected of a ∼41 kDa protein (Figure 5B). The V1G mutant showed no change in mobility when loaded with or without prior boiling, and migrated to the position expected of a protein of its molecular mass. This result indicates that V1G denatures very rapidly in the presence of SDS, and provides an explanation for its instantaneous loss of enzymatic activity in the presence of SDS (Figure 5C).

Xylanase activity profiles of all the enzymes after overnight incubation at room temperature in the presence of various SDS concentrations are shown in Figure 5C. As can be seen, the V1G mutant displayed maximum sensitivity towards SDS and became completely inactivated after a 20-min incubation in 0.5% SDS. V1F, V1L and V1D displayed maximum SDS stability. V1A and ΔV1 mutants were relatively more sensitive to increasing concentrations of SDS when compared to R-BSX.

The stability of R-BSX and its mutants at various temperatures is shown in Figure 5D. The enzymatic activity of all the mutants except V1G was found to be similar to that of R-BSX. V1G was found to be inactive after 15-min incubation at 55°C. The V1L mutant exhibited the highest residual activity at 80°C, which was in good agreement with CD experiments.

To determine the alkaline stability of R-BSX and the mutants, protein samples were incubated at various pH for 12 h to allow them to modulate structural changes with respect to pH. All the samples except the V1G mutant retained almost full activity over a complete range of pH. In contrast, the V1G mutant was almost 100% active at a pH range of 5–9, but lost 90% of its activity at pH 10 or above (Figure 5E). This loss of activity may indicate an irreversible structural change in the V1G mutant at alkaline pH (≥10).

## Discussion

The comparison of crystal structures of mesophilic and thermophilic proteins of the same family is most commonly used to understand the structural basis of thermostability. Site directed mutagenesis studies brought to light the role of salt bridges, hydrogen bonding and disulphide linkage in the thermostability of proteins [15], [2]. The comparison of the CD spectra of BSX with that of R-BSX, which contained just one additional Met, revealed a transition, although minor, at 45°C. It is possible to explain the absence of this minor transition in the R-BSX unfolding curve on the basis of increased hydrophobicity of the N-terminus due to the presence of the Met (start codon). The Met contributes to the additional hydrophobicity, which, in turn, could enhance the compact packing of the N-terminus. This conjecture was also supported by the results of the SDS-PAGE analysis. The R-BSX migrated faster than BSX, even though its molecular mass was higher due to the presence of the extra Met. A structural model of R-BSX showed the formation of two additional hydrogen bonds with the main chain and side chain of Gln2, suggesting that these may contribute to more compact packaging of the N-terminus of R-BSX.

These results suggest that the unfolding of the N-terminus is one of the first and most critical steps during the thermal unfolding of R-BSX, and that tight packing of the N-terminal region could be the mechanism by which BSX attains thermal stability. This hypothesis was further verified by substituting the surface exposed Val1 with various amino acids of differing hydrophobicity. Since the substitution of Val1 with Gly or Ala only shortens the hydrophobic chain, it is possible to interpret the difference in their thermal stability in terms of a loss of side chain atoms without raising the issue of steric hindrance around the mutant side chain. For the V1G and V1A mutants, the loss of stability can be seen as an effect of removing the methylene carbons. Removal of the first two γ-methylene carbons (Val1→Ala) decreases the thermal stability at high temperatures, but removal of the third β-methylene carbon (Val1→Gly) drastically destabilizes the V1G mutant. Our results suggest that the replacement of Val1 with Gly could affect proper docking, causing loose packing of the N-terminus, thereby facilitating its unfolding even at room temperature. Previously, Shortle et al. (1990) reported a drastic difference in stability when buried hydrophobic residues were mutated in staphylococcal nuclease (SN) [16]. The slope of the thermal denaturation curve for V1L was steeper than that of R-BSX and the rest of the mutants, which suggests that V1L shows better co-operativity in its unfolding pathway. It also indicates that regions of the protein that were previously flexible in the folded protein have now become stable due to the substitution of Leu at that position [17], [18], [19].

It has been shown that surface exposed hydrophobic residues can enhance the thermal stability of a given protein by serving as molecular “clips” or “staples” that prevent water penetration [20]. In light of these observations, the comparable thermostability of the V1D mutant and the native BSX was a bit surprising as Asp is not hydrophobic in nature. However, a comparison of the primary sequence of R-BSX with those of *Bacillus halodurans xylanase (BHX)* [21] and *Bacillus firmus xylanase (BFX)* [22] showed that in these two xylanases, Asp is present at the same position as that of Val1 in BSX. This indicates that Asp can be considered as a natural substitute for Val1 at this position, and that the aliphatic moiety of Asp is sufficient to contribute to the hydrophobic environment of the N-terminus. As described previously for trypsin [23] and human lysozyme [4], the change in hydration structure is another factor to be considered when elucidating the role of surface residues. The almost identical thermal stability of the ΔV1 mutant and R-BSX might be due to the presence of Met in the same position as that occupied by Val1 in BSX. As evident from this study, Met, being hydrophobic in nature, can provide a similar stability to ΔVal1.

The enzymatic activity of all the mutants except V1G was found to be comparable to that of R-BSX at 70°C and at pH 8.5. Optimum activity displayed by V1G was half that of R-BSX. An explanation for this observation may be provided by the native PAGE profile data, where two bands of equal intensity were detected (Figure 4A), indicating that V1G exists in two different conformations of which only one is enzymatically active.

Although the SDS-stability for many proteins has been widely discussed in the literature [20], [24], it is still not clear which physical-chemical properties contribute to this resistance. Furthermore, SDS-stability for xylanases has never been discussed before, and our study provides the first molecular and structural evidence for the SDS-stability of GH10 xylanase. SDS-PAGE analysis showed that while V1F, V1D and V1L mutants are highly stable at high concentrations of SDS, the V1G is highly susceptible to denaturation. Comparative analysis of this phenomenon with the structural features of the mutants suggested that replacement of Val 1 with more hydrophobic and bulkier residues (Phe, Asp and Leu) provides better SDS-stability, whereas the presence of small aliphatic residues (Ala and Gly) leads to the opposite effect. It further indicates that thermostable mutants of R-BSX display better resistance to SDS-denaturation compared to the less stable mutants of R-BSX. That the varying degree of SDS-stability among the R-BSX mutants correlates with their thermostability also indicates that hydrophobic surface residues provide stability at high temperatures, and suggests a correlation between thermal stability and SDS-stability. The N-terminus of R-BSX has several hydrophobic amino acids in a stretch, starting with Met (Start Codon), Val1, Pro3, Phe4, Ala5 and Trp6. Based on the structural modeling data, Asp and Phe in V1D and V1F were predicted to be involved in making additional hydrogen bonds and cation-pi interactions, respectively, which may make the N-terminal region more rigid in spite of being partially exposed. Recently, Manning et al. (2004) reported a correlation between kinetic stability and SDS-stability for many proteins with predominantly β-sheet and oligomeric structures [20]. The results presented in the present study suggest that the thermal stability and SDS-stability of R-BSX are governed by the hydrophobic nature of the substituted amino acid residue through a yet unknown mechanism. It appears that the substitution of Val1 with less hydrophobic residues is detrimental to BSX stability at high temperatures and SDS concentrations. This study also highlighted the significance of non-conserved regions in providing stability to a protein [25]. The increased SDS-stability of the V1D mutant suggests that BHX [21] and BFX [22] should retain their activity in the presence of SDS, but this needs to be verified experimentally.

The structural features responsible for BSX alkaline stability has been described previously [12]. The pH sensitivity of the V1G mutant follows the same trend as that observed in the presence of SDS, protease and at high temperature. The alkali-sensitive nature of the V1G mutant again pointed toward the fact that the N-terminus of BSX is critical for its folding and stability under various environmental stresses.

Proteins resistant to proteinase K are rare because this protease cleaves at the carboxyl side of aliphatic, aromatic or hydrophobic amino acid residues across a wide range of substrates. Markert et al. (2004) have shown that protease stable ribonuclease A can be created by changing Ala20 into Pro which changes the confirmation of the molecule [26]. In the present study, both native BSX and recombinant R-BSX were found to be resistant to proteinase K digestion. As the protease stability of BSX and R-BSX might lie in their conformation, we used proteinase K as a probe to detect any conformational changes in BSX due to conversion of Val1 to other residues. The only mutant that turned out to be susceptible to proteinase K digestion was V1G, which might be due to a change in the conformation of the protein in the N-terminal region. This was supported by the fact that V1G appeared to exist in different confirmations, as observed in the PAGE data. Our study suggests that the Val1→Gly mutation enhances local or global unfolding, specifically at the N-terminal region, which renders this mutant susceptible to temperature, SDS and proteases [20]. In other words, the N-terminus of BSX most likely plays a crucial role in its folding, which is essential for its poly-extreme stability.

In summary, our data suggest that the N-terminal region of BSX plays an important role in the thermal unfolding of the molecule and that this could be the crucial early event in this process. An exposed hydrophobic residue (Val1) was found to make a major contribution to thermal, SDS, and alkaline stability, and proteinase K resistance, thereby suggesting that these properties of BSX are somehow governed by the hydrophobic nature of the first amino acid residue. In this study, we have generated a highly thermolabile, proteinase K, alkali and SDS sensitive mutant (V1G) from a highly stable R-BSX by converting a single N-terminal exposed Val1 to Gly. These results provide insights into the structure-function relationships of xylanases under poly-extreme conditions.

## Materials and Methods

### Construction of vectors to produce recombinant xylanase (R-BSX) and its mutant enzymes in *E. coli*

The R-BSX gene (11, Genbank Acc No AF015445) encoding the mature protein (excluding the first 51aa signal sequence) was amplified from genomic DNA of *Bacillus Sp.* NG-27 using the specific primers xyla5-14 and xyla3-10, which incorporate NcoI and BamHI restriction sites, respectively. The PCR-amplified DNA fragment was cloned into complementary sites of expression vector pET14b (Novagen, Germany) to produce the recombinant plasmid pET14b xyla5-14. Various mutants of R-BSX (Table 1) were generated using a Quick Change Site-directed mutagenesis kit (Stratagene, La jolla, CA) according to the manufacturer's instruction, using pet14bxyla5-14 as a template. All mutations were verified by DNA sequencing.

### Protein expression and purification

R-BSX and its mutants were expressed in *E. coli* BL21 (DE3) and purified using a combination of anion exchange and gel filtration chromatography. A majority (>90%) of the recombinant protein and its mutants was found to be in the soluble fraction, and showed comparable biological activity with the native protein from *Bacillus*. The supernatant fraction of sonicated BL21 (DE3) cells was passed through a 0.22 μ syringe filter and loaded on to a Q-sep column connected to an ACTA purifier FPLC, and equilibrated with 50 mM Tris-Cl (pH 8.5), at a flow rate of 2 ml/min. The column was washed with 300 mM NaCl before the protein was eluted with a linear 300 mM–800 mM NaCl in a 50 mM Tris-Cl (pH 8.5) salt gradient. Protein samples were pooled and concentrated using Vivaspin Centricon (Sartorious, Germany) before being loaded on a Superdex 75 gel filtration column equilibrated with 50 mM Tris-Cl, 150 mM NaCl (pH 8.5), at a flow rate of 0.2 ml/min. Protein concentration was determined using the Bradford method [27].

### Circular Dichroism (CD)

CD measurements were carried out with a JASCO spectropolarimeter equipped with a Peltier cell holder and PTC-348 temperature controller. A protein concentration of 0.1 mg/min in 10 mM phosphate buffer (pH 7.5) and a cuvette with a 1 cm path length were used throughout. Wavelength scans were made at 20°C at a scan rate of 10 nm/min. Three scans of the same solution were averaged. The solvent spectrum was subtracted from the sample spectrum. The thermal denaturation curve was monitored by the decrease of the CD signal at 222 nm at a heating rate of 60°C/h.

### Enzyme activity assay

Xylanase activity was determined under standard reaction conditions as described previously using oat spelt xylan (Sigma-Aldrich, USA) as the substrate [7]. Unless otherwise stated, the assay was carried out at 50°C for V1G and 70°C for all other mutants including R-BSX. For thermal inactivation studies, enzymes were heated for 15 min at various temperatures and assayed for residual activity.

### Thermal unfolding detected by native PAGE

The enzyme (1 μg) was incubated for 15 min at different temperatures and then transferred immediately to 4°C to prevent any refolding. Enzymes were kept at 4°C until native-PAGE analysis.

### SDS stability

R-BSX and its mutants were incubated in various concentrations of SDS at room temperature for 12 h and residual activity was measured as described previously [11]. Xylanase activity in the absence of SDS was taken to be 100%. SDS-PAGE analysis was performed by mixing protein samples with equal volumes of sample buffer containing 0.2% SDS (final concentration) followed by incubation at room temperature for 15 min. Samples were analyzed on 12.5% PAGE gels [28].

### Proteinase K assay

The proteolysis resistance was studied using proteinase K (peptidase K, Sigma Aldrich, USA). The protein sample (1 μg) was incubated in the presence of proteinase K (0.032 units) at 28°C for 24 h and then analyzed on SDS-PAGE gels. Gels were stained with Coomassie blue.

### Alkaline stability assay

The R-BSX and its mutants were incubated at various pH for 12 h and then checked for residual activity using oat spelt xylan in Tris-Cl, pH 8.5 as a substrate under standard conditions [11].
