# Correction: The Critical Role of Partially Exposed N-Terminal Valine Residue in Stabilizing GH10 Xylanase from *Bacillus sp.*NG-27 under Poly-Extreme Conditions

**DOI:** 10.1371/annotation/aefbf6a4-298d-4173-907f-9c9a996249b2

**Published:** 2009-02-23

**Authors:** Amit Bhardwaj, Sadhu Leelavathi, Sudeshna Mazumdar-Leighton, Amit Ghosh, Suryanarayanarao Ramakumar, Vanga S. Reddy

The first author's name was spelled incorrectly. It should read: Amit Bhardwaj. This also affects the article's citation. 

